# Supplementary material for: Cost-effectiveness analysis of adding tuberculosis household contact investigation on passive case-finding strategy in Southwestern Uganda
Source: PLoS One. 2023 Dec 21;18(12):e0288761. doi: 10.1371/journal.pone.0288761 (PMC10735033; doi:10.1371/journal.pone.0288761)
Supplement: S1 File — (DOCX) [file pone.0288761.s001.docx]

**Supporting Information 1: Showing the Cost of identifying a TB case using different case-finding strategies (PCF and HHCI) from January 2020 to June 2021 across 12 facilities in Ntungamo, Rwampara, and Sheema Districts - Southwestern Uganda**

|  | Case finding modality | | | | | |
| --- | --- | --- | --- | --- | --- | --- |
| Cost category | **PCF** |  | **HHCI** |  | **PCF + HHCI** | **Data source** |
| Program Costs | **Cost US$** | **% Cost** | **Cost US$** | **% Cost** | **Costs US$** |  |
| Personnel costs |  | | | | | |
| Training | 0 | 0.00% | 12,954.00 | 21.69% | 12,954.00 | Program Activity |
| Salaries | 18,384.20 | 7.06% | 4,231.54 | 7.09% | 22,615.74 | GOU Payroll |
| Allowances and Transport | 0 | 0.00% | 23,202.38 | 38.86% | 23,202.38 | Program Activity |
| Supervision by DTLS | 0 | 0.00% | 5,269.62 | 8.83% | 5,269.62 | Program Activity |
| Phone communication | 75.28 | 0.03% | 120.28 | 0.20% | 195.56 | Program Activity |
| Printing, copying, office supplies | 3,567 | 1.37% | 4,897.00 | 8.20% | 8,464.00 | Program Activity |
| Sub Total | 22,026.48 | 8.46% | 50,674.81 | 84.87% | 72,701.30 |  |
| Total adjusted for inflation to 2021 US$ (2.16%) | **22,502.25** |  | **51,769.39** |  | **74,271.64** |  |
| Direct medical costs | | | | | | |
| Supplies & Consumables | 6,385.89 | 2.45% | 1,034.10 | 1.73% | 7,419.99 | NMS catalog |
| MTB RIF/Ultra GeneXpert | 232,029.00 | 89.09% | 8,001.00 | 13.40% | 240,030.00 | Literature |
| Sub Total Direct medical costs | 238,414.89 | 91.54% | 9,035.10 | 15.13% | 247,449.99 |  |
| Total adjusted for inflation to 2021 US$ | 243,564.65 |  | 9,230.26 |  | 252,794.91 |  |
| Total program cost | 260,441.37 | 100% | 59,709.91 | 100.00% | 320,151.29 |  |
| Total adjusted for inflation to 2021 US$ (2.16%) | 266,066.90 |  | 60,999.65 |  | 327,066.55 |  |
| Total TB cases diagnosed | 1,496.00 |  | 197.00 |  | 1693.00 |  |
| Unit cost per TB case | **174.09** |  | **303.10** |  | **477.19** |  |
| Total adjusted for inflation to 2021 US$ (2.16%) | **177.85** |  | **309.64** |  | **487.495078** |  |
|  | | | | | | |
| Direct Patient Costs |  |  |  |  |  |  |
| Average transportation for 2.4 visits @ $1.88, 2-way | 4.51 |  |  |  |  | Cost Survey **(Appendix 2: Summary results**) |
| Average transportation for one visit @ $1.88, 2-way |  |  | 1.88 |  | 6.39 | Cost Survey |
| Meals for 2.4 visits @ $2.54 | 6.10 |  |  |  |  | Cost Survey |
| Meals for one visit @ $2.54 |  |  | 1.54 |  | 7.64 | Cost Survey |
| Average caregiver costs | 4.1 |  | 0 |  | 4.1 | Cost Survey |
| Childcare/hired help/day | 3.3 |  | 1.5 |  | 4.8 | Cost Survey |
| Total | 18.01 |  | 4.92 |  | 22.93 |  |
| Indirect Patient Costs /Productivity Losses | | | | | | |
| Average total patient time lost in outpatient care (37.02hrs) |  |  |  |  |  | Cost Survey |
| Average total patient &C/giver time lost (80.15hrs) | 7.8 |  | 0.39 |  | 8.19 | Cost Survey |
| Average total patient &C/giver time lost in HHCI (2 hrs.) |  |  |  |  |  |  |
| Min. wage hourly rate in Uganda ($0.15) |  |  |  |  |  | Cost Survey |
| Total | 25.81 |  | 5.31 |  | 31.12 |  |
| Patient total cost adjusted for inflation to 2021US$ (2.16%) | **26.37** |  | **5.42** |  | 31.79 |  |

1. *Program costs include administration, transport, communication & health personnel*
2. *Direct medical costs include MTB RIF/Ultra GeneXpert & Supplies & Consumables.*
3. *Total patient and caregiver costs include direct (transportation& meals) and Indirect costs
   (productivity/wages lost)*

*Estimated total per-patient costs are a summation of program, direct medical, and total patient-caregiver
Costs estimate*
