# Supplementary figures and images for: Cost-effectiveness analysis of adding tuberculosis household contact investigation on passive case-finding strategy in Southwestern Uganda
Source: PLoS One. 2023 Dec 21;18(12):e0288761. doi: 10.1371/journal.pone.0288761 (PMC10735033; doi:10.1371/journal.pone.0288761)

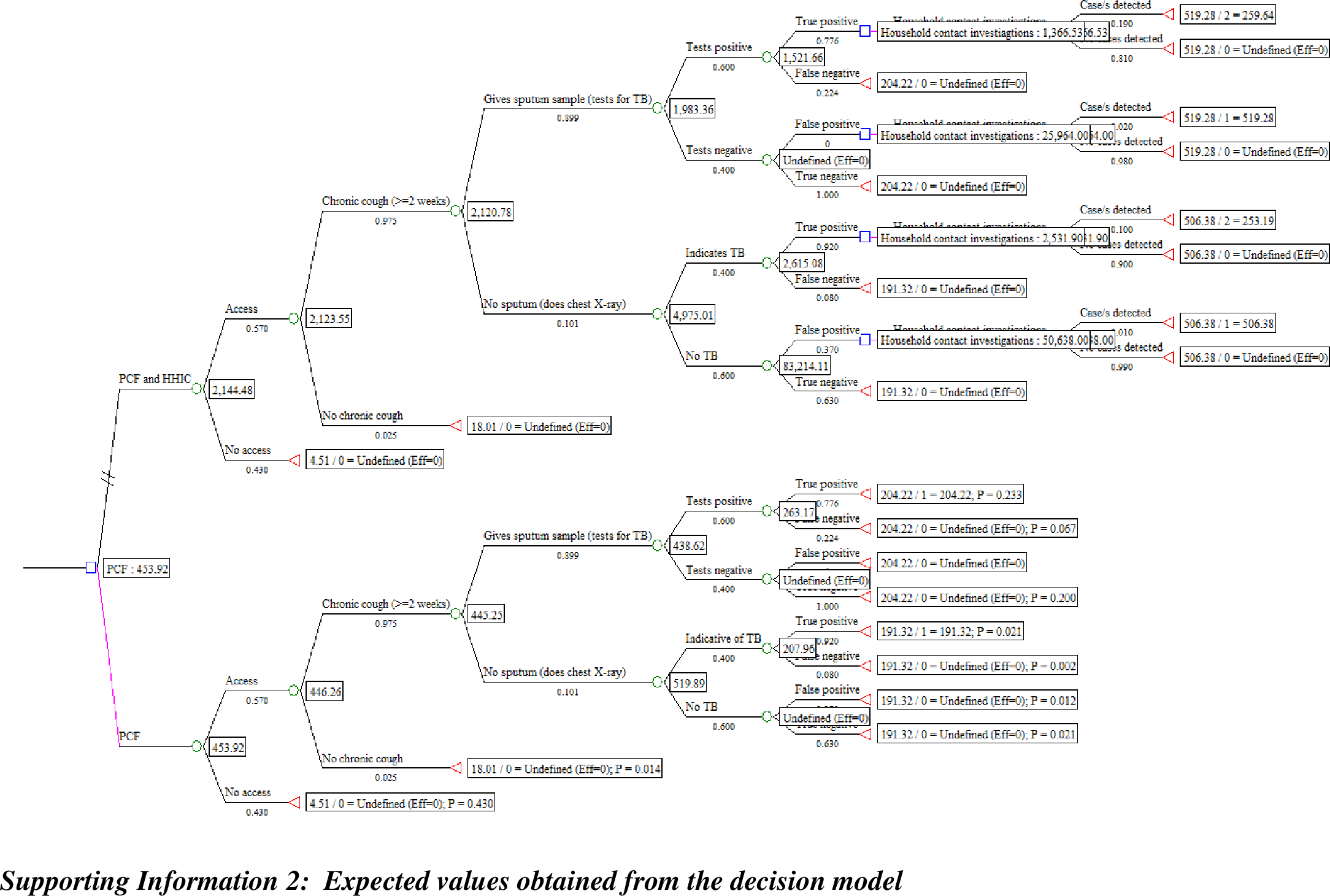

Supplement: S2 File — (TIF) [file pone.0288761.s002.tif]
