# Supplementary material for: Cost-effectiveness analysis of adding tuberculosis household contact investigation on passive case-finding strategy in Southwestern Uganda
Source: PLoS One. 2023 Dec 21;18(12):e0288761. doi: 10.1371/journal.pone.0288761 (PMC10735033; doi:10.1371/journal.pone.0288761)
Supplement: S3 File — (PDF) [file pone.0288761.s003.pdf]

SUPPORT DOCUMENT 1

Supporting 1: Patient Survey Questionnaire

Health facility and community TB patient costs survey form

This interview guide is prepared to collect patient information on costs and caregiver information from a societal perspective.

Part 1 ID # Unit TB number

Health facility name where the client was diagnosed with TB

Qn.1. Sex 01 = Male, 02 = Female

Qn.2. Age (years) Use code 00; I don't know

Qn.3.Residence 01 = Ntungamo, 02 = Rwampara, 03 = Sheema

Qn.4. Are you receiving TB treatment? 01 = Yes, 02 = No

Qn..5. When did you start your TB treatment? ***Write date/month/year in the boxes***

Qn.6.How long have you been taking TB treatment? Use code 00; I don't know

Weeks

Month

Costs information: The questions below refer to visits associated with your TB diagnosis

Qn.7. How often did you travel to this health facility to evaluate your current TB episode (Here, I mean when you had the sputum and other TB tests)? Use code 00; I don't know.

Number of times

Qn.8. How did you travel to the health facility for diagnostic evaluation of your current illness?

01 = Public means, 02 = Hired car, 03 = Worked/Foot, 04 = Boda boda, 05 = Personal car, 06 = Other, Specify…………………………

Qn.9. How much money did it cost you to travel from your home to the health facility for the TB evaluation (One-way fare)? Uganda Shillings

Qn.10. How much time did you spend traveling to the health facility for diagnostic evaluation visits?

Hours Minutes

Qn.11. How much time did you spend waiting in line to see the health provider at the diagnostic evaluation visit? Hours Minutes

Qn.11. a. Did you have a chest X-ray taken 01 = Yes 02 = No

Qn.11. b. If "YES," how much did you pay for the chest X-ray? Uganda Shillings

Qn.12.Did you spend money on food/snacks/drinks while on any diagnostic evaluation health facility visit? 01 = Yes, 02 = No

Qn.13. If yes, estimate how much money you spent on food/drinks/snacks while on any diagnostic visits for your current illness? Uganda Shillings

Qn.14. Do you think you would have spent the same money on food/drinks/snacks without attending diagnostic health facility visits? 01 = Yes, 02 = No

Qn.15. Are you currently employed? 01 = Yes, No = No

Qn.16. What is your occupation?

Qn.17. What is the average monthly earning from all your jobs?

Uganda Shillings

Qn.18. Did you take days off from work to come to the health facility to evaluate your current illness? 01 = Yes, 02 = No, 00 I don’t remember

Qn.19. How much time do you spend while seeking medical evaluation at the health facility? Hours Minutes

00 = I don't remember

Qn.20. Do you hire someone for the short term to manage your business when taking diagnostic evaluations?

01 = Yes, 02 = No

Caregiver Information

Qn.21.Did you have any person accompanying you to any diagnostic evaluation visits?

01 = Yes, 02 = No

Qn.22. If yes, how many people accompanied you on the diagnostic evaluation visits?

Qn.23. How many of your caregivers were employed?

Qn.24. Did you specifically hire someone to take care of your children or your home while you were away attending the health facility visits to diagnose your current illness?

01 = Yes, 02 = No

Qn.25. How much roughly did you pay this person when you were away for health facility visits? Uganda Shillings

We appreciate you so much for providing us with the information to serve you better.

Interviewer Initials Date of completion

Reviewer Initials Date of completion
