# Supplementary material for: Cost-effectiveness analysis of adding tuberculosis household contact investigation on passive case-finding strategy in Southwestern Uganda
Source: PLoS One. 2023 Dec 21;18(12):e0288761. doi: 10.1371/journal.pone.0288761 (PMC10735033; doi:10.1371/journal.pone.0288761)
Supplement: S5 File — (PDF) [file pone.0288761.s005.pdf]

Supporting Document 3: Health Provider Costing Questionnaire

Costing Questionnaire for Tuberculosis case detection

This interview guide is prepared to collect expert evidence from the health care professionals (Medical doctors, Clinical officers, nurses, laboratory personnel, cough monitors/T.B., the linkage facilitators) working on Tuberculosis infection prevention, care, treatment, and control.

This guide contains the following sub-sections:

A. General facility information

B. Health professional time- labor cost

C. Health professional time - across the TB case finding

D. Supplies and laboratory equipment/Tests typically used during TB case finding health service provision

E. Training Cost (Basic training) for medical officers, Nurses, TB linkage facilitators, and other healthcare professionals

**A General Facility Information**
Data collector: (1) A1-A5 to fill in before interview except for A6 (2) For A6, all persons contacted to fill this questionnaire should be listed; indicate whether the respondent interviewed in the last column. (3) In general, data collectors should refer to the data Collector Guide for terms and definitions that may not be clear.

A1	Name of Health Facility				
A2	District				
A3	Type of Health facility		Government ☐ PNFP ☐		
A4	Level of Health Facility		III. ☐ IV ☐ Hospital ☐		
A5	Total Number of Health workers in the facility				
A6	1. Respondent name	2. Carder	3. Unit	4. Tel	5. Interviewed (Y/N)
1					
2					
3					
4					

B. Health Professional time labor cost

	Please provide the following information for the staff that works at your facility in providing TB service on the facility payroll:								
	1. Staff Type/carder (please also include the level)	2. # of staff working at this carder level	3. Staff monthly salary/ Pay grade, scale	4. Organization that pays	5. Total Days worked per month on any service	6. Total days worked per month TB contact tracing activity	7. Average min Taken screening and detection per client	8. Hrs Total days worked per month on TB Screening and case detection	8. Remarks
1	Certificate Nurse/Midwife								
2	Assistant NO								
3	Nursing officer								
4	Clinical officer								
5	Lab Assistant								
6	Lab Tech								
7	TB Linkage/Peer								
8	Medical Officer								
9	Other professionals								

C. Health professional time - across the TB case finding

**Staff Carder**	# in TB clinic	Average Hr to screened patient PCF	# patient screened PCF	# diagnosed PCF	# contacts screened	#contact diagnosed	# Household visited months	Average Hr spent on household per patient
Nurse/Midwife								
Assistant NO								
Nursing Officer								
Clinical Officer								
Lab Assistant								
Lab Tech								
T.B. L.F./Peer								
Medical Officer								
Other Professional								

D. Supplies and laboratory equipment/Tests typically used during TB case finding health service provision

SN.	Would you please list the supply items used in the TB diagnosis	Unit of measure	Unit Price as per NMS	Notes/Remarks
1				
2				
3				
