# Supplementary material for: Cost-effectiveness analysis of adding tuberculosis household contact investigation on passive case-finding strategy in Southwestern Uganda
Source: PLoS One. 2023 Dec 21;18(12):e0288761. doi: 10.1371/journal.pone.0288761 (PMC10735033; doi:10.1371/journal.pone.0288761)
Supplement: S6 File — (PDF) [file pone.0288761.s006.pdf]

Supporting document 4: Data Extraction Tools for Household and Passive Case Finding

The data extraction tools for a Household Contact Investigation tool and Passive case findings for the facility

Form 1. A Contact tracing investigation tool for extraction of household contact investigation data from the contact tracing register

Contact ID#	Age	Sex	HIV status	Contact screened for TB.	Presumed for TB.	Diagnosed with TB (New & Relapsed)	Disease Class	Diagnostic method	Linked to treatment

Form 1. B for extraction of an Index case data from the contact tracing register

Index ID#	Age	Sex	HIV status	Disease class	Diagnostic method	On TB treatment	Total number of contact/s	Total Contact/s followed

Form 1. C for extraction of PCF data from DHIS2 (1st JAN 2020 – 30th June 2021)

Health facility Name	Number of clients screened	Number of presumptive TB cases identified	Number of TB cases diagnosed (New and Relapse)	Testing method	Disease class	Age	Sex	The number started on treatment
