# Supplementary material for: Cost-effectiveness analysis of adding tuberculosis household contact investigation on passive case-finding strategy in Southwestern Uganda
Source: PLoS One. 2023 Dec 21;18(12):e0288761. doi: 10.1371/journal.pone.0288761 (PMC10735033; doi:10.1371/journal.pone.0288761)
Supplement: S7 File — (PDF) [file pone.0288761.s007.pdf]

Supporting document 5: Ethical Clearance and approvals

GULU UNIVERSITY

P.O. Box 166, Gulu (U)

Tel: +256 471 432 096
Fax: +256 471 432 913
Mob: +256 772 305 621
+256 776 812 147

Website: www.qq.ac.ug
Email: qurec@qu.ac.ug

RESEARCH ETHICS COMMITTEE

16/11/2021

To: Dickens Odongo

Lira University
0758246030

Type: Initial Review

Re: GUREC-2021-100: Cost-Effectiveness of adding Tuberculosis Household Contact Investigation on Passive Case Finding strategy in South Western Uganda, PDF, 3.0, 2021-11-12

I am pleased to inform you that at the 77th convened meeting on 23/09/2021, the Gulu University REC committee meeting, etc. voted to approve the above-referenced application.
Approval of the research is for the period of 16/11/2021 to 16/11/2022.

As Principal Investigator of the research, you are responsible for fulfilling the following requirements of approval:

1. All co-investigators must be kept informed of the status of the research.
2. Changes, amendments, and addenda to the protocol or the consent form must be submitted to the REC for review and approval for the activation of the changes.
3. Response of unanticipated problems involving risks to participants or any new information which could change the risk-benefit: ratio must be submitted to the REC.
4. Only approved consent forms are to be used in the enrollment of participants. All consent forms signed by participants and/or witnesses should be retained on file. The REC may conduct audits of all study records, and consent documentation may be part of such audits.
5. Continuing review applications must be submitted to the REC eight weeks before the expiration date of 16/11/2022 to continue the study beyond the approved period. Failure to submit a continuing review application in a timely fashion may result in suspension or termination of the study.

6. The REC application number assigned to the research should be cited in any correspondence with the REC of record.
7. You are required to register the research protocol with the Uganda National Council for Science and Technology (UNCST) for final clearance to undertake the study in Uganda.

The following is the list of all documents approved in this applicatio by Gulu University REC

GULU UNIVERSITY
INSTITUTIONAL REVIEW COMMITTEE
APPROVED UNTIL
★ 16 NOV 2022 ★
FACULTY OF MEDICINE
P. O. Box 166, Gulu

No.	Document Title Language	Version Number	Version Date
1	Informed Consent forms	English PDF, 3.0	2021-1 1-12
2	Informed Consent forms	English PDF, 3.0	2021-1 1-12
3	Informed Consent forms	RunyankolPDF, 3.0	2021-1 1-12
4	Data collection tools	English PDF, 3.0	2021-1 1-12
5	Protocol	English PDF, 3.0	2021-1 1-12
6	COVID 19 SOPs	English	

GULU UNIVERSITY
INSTITUTIONAL REVIEW COMMITTEE
APPROVED UNTIL
★ 16 NOV 2022 ★
FACULTY OF MEDICINE
P. O. Box 166, Gulu

Yours Sincerely

Dr. Gerald OBAI

Appendix 8: Patient or caregiver informed consent – English Version, 3.0

Title of the study: Cost-Effectiveness of adding Tuberculosis Household Contact Investigation on Passive Case Finding strategy in South Western Uganda

Investigator(s): Odongo Dickens

Institution(s): Lira University

Introduction

I am a student at Lira University pursuing a Master of Public Health and conducting research on the costs of Tuberculosis diagnosis. This informed consent explains the study to you. After the survey presentation, I will answer any questions you may have, and after you have decided to participate in the survey, you will be requested to sign a consent, and a copy availed you to keep. Your participation in this study is voluntary, and if you choose to withdraw from the study at any point, your information will be deleted and not used at any point in the data analysis and write-up

A brief description of the sponsors of the research project

It is academic research, and there is no sponsor for the project.

Purpose:

The study compares the cost-effectiveness of active household contact investigation and passive case finding in rural and periurban settings in South Western Uganda. The findings will provide evidence for informed policy decisions and improved practice of TB case findings. The study findings and recommendations shall be helpful to all stakeholders, including local governments, MoH, and NGOs involved in TB prevention and control. Last but not least, it is an academic research study as partial fulfillment of the requirement for the Master of Public Health Lira University award. It shall add to the existing pool of knowledge on cost-effectiveness studies in Uganda.

Procedures:

You will be asked several questions about the costs you spent while coming and waiting for the result of your current TB diagnosis. You are free to decline to answer any questions you do not want to answer.

I will read the questions to you about transport fees, food, and drinks expenses. Other indirect costs you incurred during travel time, waiting time, and absence from work will be asked. You will also be asked about the costs if you come with a caregiver.

Who will participate in the study?

You have been chosen to participate in this study because you were diagnosed and getting Tuberculosis treatment from this facility. The interview will last for approximately 30 to 45 minutes, and three of your colleagues will also participate in a similar study.

Risks/discomforts:

There is no foreseeable risk of harm or discomfort that will arise from your participation in this study. The only risk or discomfort will be the inconvenience in terms of time spent during the interview.

Benefits:

By taking part in this research study, you will help the study personnel to identify TB case finding methods with the most nominal costs and are easy to implement. You will be given health education on your treatment adherence and referred to a doctor if you are not feeling well due to the side effects of your medications.

The study information will also help the Ministry of Health officials in Uganda plan better TB/ HIV control programs for many similar communities in Uganda, which may benefit society in the future.

Confidentiality:

Your identity will not be revealed to anyone, as I shall only use codes to identify participants. Information obtained will only be accessible by the research team. The principal investigator will use a password to protect soft copies of the data and hard documents kept under lock and key. Only the principal investigator will have access to your confidential information.

Alternatives:

You do not have to participate in this study if you are not interested, and if you choose to withdraw from the study, your information will be deleted and not used at any point in the data analysis and write-up. You will not lose any benefit in case of no participation.

Costs:

There will not be any additional cost incurred as a result of participating in this study.

Questions:

If you have any questions related to the study as a research participant, you can contact the principal investigator, Odongo Dickens, on telephone number 0774 377545 or via email at dodongo1980@gmail.com

Statement of voluntariness:

Participation in the research study is voluntary, and you may join of your own free will. You have a right to withdraw from the study at any time without penalty. If you have any issues about your rights and participation in the study, don't hesitate to get in touch with the Chairperson, Gulu University Research Ethics Committee, Dr. Gerald Obai Tel: No.,

0772305621; email: lekobai@yahoo.com/lekobai@gmail.com; or the Uganda National Council

for Science and Technology, on plot 6 Kimera Road, Ntinda, Kampala on Tel 0414705500.

Statement of consent

.... has described to me what is going to be done, the

risks, the benefits involved, and my rights as a participant in this study. I understand that my decision to participate in this study will not affect me in any way. In the use of this information, my identity should be concealed. I am aware that I may withdraw during the study, and my information will be deleted and not used at any point in the data analysis and write-up. By signing this form, I understand that I do not waive any of my legal rights. Still, I merely indicate that I have been informed about the research study in which I voluntarily agree to participate. I will be provided with a copy of this form.

Name ………………………… Signature of participant……………………. Date………………

Name…………………………. Signature of interviewer……………………. Date………………

documents approved in this applicatio

GULU UNIVERSITY
INSTITUTIONAL REVIEW COMMITTEE
APPROVED UNTIL
★ 16 NOV 2022 ★
FACULTY OF MEDICINE
P. O. Box 166, Gulu

Appendix 9: Patient or caregiver informed consent –Runyankole Version 3.0

EKIHANDIKO KYOKUHAMYA OKWIKIRIZA ABWA OMURWAIRE NO'MUJNJABI

Omutwe Gwo'kurondora: Okugyeragyeranisa enkora eshorongire yokukyebera abantu abokutura omumaka gabantu abahamibwe kuba baine oburwaire bwa'kakonko nan'abo abakutekatekwaho kuba baine oburwaire nibwo bumwo, omu ntura yekyaro nomutown za'mashuma go'buregyerwa eizoba bwa Uganda.

Karondozi: Odongo Dickens

Institution(s): Lira University

Entandikiriro

Ndi Omwegi wa yunivasite wa Lira ninshoma diguri yaheiguru omu byamagara kandi ndiyo ninkora okurondoza oburemezi bwo'kuzoora oburwaire bwa'kakonko. Ekihandiko eki nikikushoborora aha kurondoza oku, nokushaba okwikiriza kwawe kukwetabamu. Okurondoza oku kwabaho, ebibuzo byona ebi orabase kugarukamu bwanyuma yo'kwikiriza kwetabamu, noshabwa kuhamya orikuta omukono nainga ekinkumu aha kihandiko eki ku waikiriza kwetabamu otagyemirwe. Okwetaba omu kurondoza oku kyemereire aha kukunda kwawe kandi oine obugabe kurugamu obwire bwona kandi amakuru n'ebiri kukukwataho byona bisazibwemu kandi bitejunizibwa omu muringo gwona gwo'kucencura nainga kuhandinka

Okushorera bugufu aha byokushagika okurondoza oku

Okurondoza oku nikuza kwejunisibwa omu byokwega kandi tihariho muntu wena nainga ekitongore kyona ekirikushagika.

Ekigyendererwa

Okurondoza oku nikugyeragyeranisa enkora eshorongire yokukyebera abantu abokutura omumaka gabantu abahamibwe kuba baine oburwaire bwa'kakonko nan'abo abakutekatekwaho kuba baine oburwaire nibwo bumwe, omu ntura yebyaro nomumatown ga'mashuma go'buregyerwa eizoba bwa Uganda. Ebirarugye omukurondoza nibiza kworeka obuhame bwokugyenderaho kuretaho enkora nungi yo'kurondora oburwaire bwa'kakonko. Ebirarugye omu kurondoza oku nibiza kuyamba abantu bekicweka eki, ebebembezi ba gavumenti zebyanga, minisiture yebyamagara hamwe nebitongore bitari bya gavumenti ebiri kwetaba omukutangira no'kuzibira oburwaire bwa'kakonko. Ekyaha muheru nokukumanyisa ngu oku'condoza oku nikimwe aha byetago

byo’kutunga diguri ya’ihuguru omu byamagara kuruga omu yunivasite ya Lira. Eki nikiza kwongyera aha kumanya no’kuha obuhereza bushorongire omu Uganda.

Enkuratiriro

Noza kubuzibwa ebibuzo byingi bikwatiraine na sente ezi wakozeise omu’kwaija n’okutegyereza ebyarugire omu kukyebeza endwara y’akakonko eyoyine hati. Oine obugabe kwanga kugarukamu ebibuzo ebi otakwenda. Ninza kukushomera ebibuzo bikwatiraine n’ebicwero by’entambura, ebyokurya, ebyokunywa, n’endijo nshohoza eyakwihikireho omu’kutambura, okutegyereza nobutaza aha murimo gwawe. Kandi nabwe nituza kukubuza aha nshohoza eyiwakozeise kuwakuba waizire n’omujanjabi ahairwariro.

Noha orayetabe omu kurondoza oku?

Otoreinwe kwetaba omu kurondoza oku abwokuba okazorwamu kandi notunga emibazi y’oburware bw’ankonko ah’irwariro eri. Okubuzibwa oku nikuza kumara edakika ziri aha gati 30 na 45, kandi abandi batahi bangye bashatu nabo nibiza kwetaba omu’kurondoza okuri nkoku.

Obugyengye

Tihariho kirikutekwatekwa kuretaho obugyengye ahabw’okwetaba kwawe omu kurondoza oku nainga okuteganisibwa aha bikwatiraine n’obwire bworamare omu’kubuzibwa.

Ebyomugasho

Okwetaba omukurondoza oku, nikiza kuhwera abashaho kumanya emiringo yo’kuzora oburwaire bwa’kakonko aha nshohoza nkye kandi kyorobi no’kuta omu’nkora. Noza kuhebwa okwegyesibwa aha’byamagara no’kuhamira aha’kuraguza kandi oyehorezibwe aha mushaho waba otakuhuriragye aha’bwemibazi eyo’kumira. Amakuru go’kurondoza nigaza kuyamba abakuru omu minisiture ye’byamagara kuretaho entekateka nungu zo’kutangira obu’rwaire bwa’kakonko na sirimu bicweka byona bya Uganda no’kugasira abantu omu biro byo’mumaisho.

Ebihama

Ebye’ndanga muntu yawe tibirashurirwe muntu wena, ahabwo’kuba ninza kukozesa za kodi kumanya abarayetabe omu kurondoza oku. Amakuru agararugye omu’kurondoza nigaza kurebwa tiimu yaba’karondozi yonka. Ebihandiko byona ebyine akakwate nokurondoza oku nibiza kubikwa omumwanya gukusibwa. Omucondoza omukuru wenka niwe arabe naikirizibwa kwihika aha’makuru age’kihama.

Ebindi

Otakayetaba omu'kurondoza oku waba otari kwenda kandi wakunda kuruga omu kurondoza oku, amakuru gawe nigaza kusazibwamu kandi gatejunizibwa omu muringo gwona gwo'kucencura nainga kuhandinka.

Enshohoza

Tiharabeho nshohoza yona ahabwo'ketaba omukurondoza oku.

Ebibuzo

Waba oine ekibuzo kikwatiraine no'kurondoza oku, nk'omwe omubakwetabiremu, nobasa kwihikirira oyembeire okurondoza oku, omw. Odongo Dickens aha namba ye'simu egi: ***0774 377545*** or nainga aha mukutu ogukugaita abantu orikurabira aha: ***dodongo1980@gmail.com***

Ebyo'kwikiriza kwetaba omukurondoza

Kiri aha'kwikiriza kwawe kwejumbira omu kurondoza oku. Oine obugabe kuruga omu kurondoza oku obwire bwona obuwakunda hataine okukwatsire aha mukono. Waba oine ekibuzo kyona ekikwatireine nobugabe bwawe omukurondoza oku, otatina kwihikirira orikwebembera akakiko akajunanizibwe ne'mitwarize yo'kurondoza aha yunivasite ya Gulu **Dr. Gerald Obai** aha namba ye'simu egi: **0772305621**; nainga aha mukutu ogukugaita abantu orikurabira aha: **lekobai@yahoo.com/lekobai@gmail.com**; nainga ohikirire Akakiiko ka Uganda yona ahabwe byasayansi na tekinorogiya, aha kibanja 6 oruguto rwa Kimera, Ntinda, Kampala aha namba ye'simu egi: **0414705500**.

Okuhamya okwikiriza kwangye

.... yanshoborora ibikuza Kukorwa, ebirikuretaho obugyengye, eburungi birimu, nobugabe bwangye kwejumbira omu kurondoza oku. Namanya ngu okucwamu kwejumbira omu kurondoza oku tikeine kabi omumuringo gwona ahari'nye. Kandi Namanya ngu omukukoresa amakuru aga, ebirikunkwataho byona byine kusherekwa. Nimanya kurungi ngu nimbasa kuruga omu kurondoza oku obwire bwona, amakuru gangye gasazibwemu kandi gatejunizibwa omu muringo gwona gwo'kucencura nainga kuhandinka. Okuta omukono aha kihandiko eki, Nimanya ngu tindikwihaho bugabe bwangye obu'kugyendera ahamateeka. Nobu biri bityo, ninyoreka ku namanyisibwa aha bikwatiraine noku'rondoza oku kandi naikiriza ntagyemirwe kukwetabamu, ninza kuhebwa copy yekihandiko eki.

Ninye ………………………… Omukono/Ekinkumu……………………. Ebiro………………

Owabuza……………………… Omukono/Ekinkumu ………………… …...Ebiro………………

documents approved in this applicatio

GULU UNIVERSITY
INSTITUTIONAL REVIEW COMMITTEE
APPROVED UNTIL
★ 16 NOV 2022 ★
FACULTY OF MEDICINE
P. O. Box 166, Gulu

Appendix 10: Healthcare providers informed consent 3.0

Title of the study: Cost-Effectiveness of adding Tuberculosis Household Contact Investigation on Passive Case Finding strategy in South Western Uganda

Investigator(s): Odongo Dickens

Institution(s): Lira University

Introduction

I am a student of Lira University pursuing a Master of Public Health and conducting research on the costs of Tuberculosis diagnosis. This informed consent explains the study to you. After the survey presentation, I will answer any questions you may have, and after you have decided to participate in the survey, you will be requested to sign a consent, and a copy availed you to keep. Your participation in this study is voluntary, and if you choose to withdraw from the study at any point, your information will be deleted and not used at any point in the data analysis and write-up

A brief description of the sponsors of the research project

It is academic research, and there is no sponsor for the project.

Purpose:

The study compares the cost-effectiveness of active household contact investigation and passive case finding in rural and periurban settings in South Western Uganda. The findings will provide evidence for informed policy decisions and improved practice of TB case findings. The study findings and recommendations shall be helpful to all stakeholders, including local governments, MoH, and NGOs involved in TB prevention and control. Last but not least, it is an academic research study as partial fulfillment of the requirement for the Master of Public Health Lira University award. It shall add to the existing pool of knowledge on cost-effectiveness studies in Uganda.

Procedures:

You will be asked about your expert experiences from the health care professionals (Medical doctors, Clinical officers, nurses, laboratory personnel, cough monitors/T.B., the linkage facilitators) working on Tuberculosis infection prevention, care, treatment, and control. You are free to decline to answer any questions you do not want to answer. I will read the questions about health professional time, labor costs across the TB finding, supplies, equipment, Tests, training costs.

Who will participate in the study?

You have been chosen to participate in this study because you are a knowledgeable stakeholder in TB healthcare service delivery planning, implementation, and evaluation. The interview will last for approximately 45 - 60 minutes, and eleven of your colleagues will also participate in a similar study.

Risks/discomforts:

There is no foreseeable risk of harm or discomfort that will arise from your participation in this study. The only risk or discomfort will be the inconvenience in terms of time spent during the interview.

Benefits:

By taking part in this research study, you will help the study personnel to identify TB case finding methods with the most nominal costs and are easy to implement. The study information will help the Ministry of Health officials and Local Government and implementing partners in Uganda plan better TB/ HIV control programs for many similar communities in Uganda, which may benefit society in the future.

Confidentiality:

Your identity will not be revealed to anyone, as I shall only use codes to identify participants. Information obtained will only be accessible by the research team. The principal investigator will use a password to protect soft copies of the data and hard documents kept under lock and key. Only the principal investigator will have access to your confidential information.

Alternatives:

You do not have to participate in this study if you are not interested, and if you choose to withdraw from the study, your information will be deleted and not used at any point in the data analysis and write-up. You will not lose any benefit in case of no participation.

Costs:

There will not be any additional cost incurred as a result of participating in this study.

Questions:

If you have any questions related to the study as a research participant, you can contact the principal investigator, Odongo Dickens, on telephone number 0774 377545 or via email at dodongo1980@gmail.com

Statement of voluntariness:

Participation in the research study is voluntary, and you may join at your own free will. You have a right to withdraw from the study at any time without penalty. If you have any issues about your rights and participation in the study, don't hesitate to get in touch with the Chairperson, Gulu University Research Ethics Committee, Dr. Gerald Obai Tel: No.,

0772305621; email: lekobai@yahoo.com/lekobai@gmail.com; or the Uganda National Council

for Science and Technology, on plot 6 Kimera Road, Ntinda, Kampala on Tel 0414705500.

Statement of consent

.... has described to me what is going to be done, the

risks, the benefits involved, and my rights as a participant in this study. I understand that my decision to participate in this study will not affect me in any way. In the use of this information, my identity should be concealed. I am aware that I may withdraw during the study, and my information will be deleted and not used at any point in the data analysis and write-up. By signing this form, I understand that I do not waive any of my legal rights. Still, I merely indicate that I have been informed about the research study in which I voluntarily agree to participate. I will be provided with a copy of this form.

Name ………………………… Signature of participant……………………. Date………………

Name…………………………. Signature of interviewer……………………. Date………………

documents approved in this applicatio

GULU UNIVERSITY
INSTITUTIONAL REVIEW COMMITTEE
APPROVED UNTIL
16 NOV 2022
FACULTY OF MEDICINE
P. O. Box 166, Gulu

Appendix 11: Prevention and Control SOPs for COVID -19 Version 3.0

Measures for the prevention and control of the risk of the spread of COVID-19 during the implementation of research

Study Title: Cost-Effectiveness of adding Tuberculosis Household Contact Investigation on Passive Case-Finding strategy in South Western Uganda

Principal Investigator (s): Odongo Dickens

Introduction:

The novel Coronavirus is transmitted from human to human through droplets and direct or close personal contact with an infected individual

Novel Coronavirus signs of infection include respiratory symptoms, fever, cough, shortness of breath, and breathing difficulties. In more severe cases, the disease can cause pneumonia, severe acute respiratory syndrome, kidney failure, and even death

Implementation of the prevention and control measures

In implementing the research activities, the investigators are committed to ensuring the safety of their research team, research participants, and communities where the study will be conducted. The study team undertakes to comply with the Standard Operating Procedures issued by the Ministry of Health and presidential directives to mitigate against the risk of infection of COVID-19, rapidly detect and effectively respond to any COVID-19 case that may occur in the process of carrying out the study, screening, face covering physical distancing, and good hand hygiene.

Procedures to be followed during the implementation of the research.

Before data collection and training

1. **Health guidance:** There will be training sessions dedicated to COVID-19 sensitization and awareness to equip the research team with knowledge of signs and symptoms of the COVID19 and preventive measures such as hand hygiene before placing and removing the mask as storage of the show, social distancing. The research team will circulate notices, posters, charts on common signs and symptoms of COVID-19 as provided by the Ministry of Health and develop and use existing plans for the appropriate referral pathway for identified and suspected cases.
2. **Personal Screening:** All research assistants and, participants, and other individuals" engaged in research activities will take the daily temperature for fever; screening for temperature will be

carried out during planning meetings, training, community outreach. Any individual with 19 symptoms will be withdrawn and referred to the COVID - 19 task for further assessment and management. The withdrawn individual will be allowed to return only if they present a valid certification of' their COVIJ)-19 status showing a negative result.

3. **Wearing face coverings**: All researchers, research assistants, and any other individuals engaged in research activities that require interaction with fellow researchers, research participants, or the community member will have to wear a face mask that covers the mouth and nose at all times. The study will provide face masks for individuals who do not have them.
4. **Physical Distancing**: There will be social distancing during the training, meetings, and community outreach of at least 2 meters. During breaks, the team will not be allowed to congregate in common areas.
5. **Good hygiene**: All team members will be required to wash their hands or use hand sanitizers before and after entering the training room and other common areas. Hand-washing equipment shall be supplied and made available at all times. All surfaces and equipment shall be sanitized frequently.

During data collection;

l .**Personal Protective Equipment (P PE):** All research assistants involved in tracing respondents and in-person interviews and research participants will use PPE, including a properly fitted face mask. Enumerators/Researchers and research participants will wash hands with soap and water or use hand sanitizer before the interview. Handshakes and hugging are prohibited. The physical distancing of at least 2 meters in all research-related activities shall be observed.

2. **Focus group discussions**: The research team shall carry educational materials on the prevention of COVID-19 in a language understood by the participants. These materials shall also have visual images to support understanding. Enumerators/Researchers and research participants will wash hands with soap and water or use hand sanitizer before the interview. Screening for temperature will be carried out before conducting meetings
3. **Consent participants** or Research assistants will observe social distancing when the participant. Enumerators/Researchers and research participants will wash hands with soap and water or use hand sanitizer before the interview. Participants will then be sensitized to covid-19 and presented with preventive measures.
4. **Equipment sanitization**: shared devices used to record or capture data shall be sanitized regularly.

During Dissemination/Community engagement

l. Community Engagement: The research team shall carry educational materials on the prevention of COVID-19 in a language understood by the participants. These materials shall also have visual

images to support understanding. Enumerators/Researchers and research participants will wash hands with soap and water or use hand sanitizer before the interview. Screening for temperature will be carried out before conducting community engagements.

5. **Equipment sanitization**: shared devices used during community engagements shall be sanitized regularly.

The preventive and control measures will continuously be reviewed based on new information and guidelines communicated by the Ministry of Health

GULU UNIVERSITY
INSTITUTIONAL REVIEW COMMITTEE
APPROVED UNTIL
16 NOV 2022
FACULTY OF MEDICINE
P. O. Box 166, Gulu

Appendix 12: DHOs and Facilities Approvals

Kabwohe HC-IV

LIRA UNIVERSITY

LIRA UNIVERSITY
THE BEACON

P.O. Box 1035
Lira, Uganda
Tel: +256 414-694716
Web: www.lirauni.ac.ug
Email: health.faculty@lirauni.ac.ug.

FACULTY OF HEALTH SCIENCES
DEPARTMENT OF PUBLIC HEALTH

6th December, 2021

Office of the District Health Officer ... 17 JAN 2022 ... DISTRICT

In charge, Accord this MPH student necessary support.

TO WHOM IT MAY CONCERN

RE: ODONGO DICKENS **REG. NO:** 19/HDO3/U/0109

This is to introduce to you the above-named person, a second-year student of Lira University pursuing Master of Public Health (MPH) Degree.

In partial fulfillment of the requirements for the award of Masters' Degree, he/she is carrying out research titled "Cost -Effectiveness of adding Tuberculosis Household Contact Investigation on Passive case finding strategy in South Western Uganda

The purpose of this letter is therefore to request you to allow him/her to access information/data from your organization. The data collected will be for academic purposes only.

We shall highly appreciate any assistance accorded to him/her during the course of the fieldwork.

Yours sincerely,

LIRA UNIVERSITY
P.O BOX 1035, LIRA
06.12.2021
HEAD OF DEPT.
PUBLIC HEALTH

MS AKELLO ANNE RUTH
HEAD OF DEPARTMENT, PUBLIC HEALTH

KABWOHE HEALTH CENTRE IV
[illegible]
18 JAN 2022
INCHARGE
P.O BOX [illegible] KABWOHE

Received

Kyangyenyi HC III

LIRA UNIVERSITY

LIRA UNIVERSITY
THE BEACON

P.O. Box 1035
Lira, Uganda
Tel: +256 414-694716
Web: www.lirauni.ac.ug
Email: health.faculty@lirauni.ac.ug.

FACULTY OF HEALTH SCIENCES

DEPARTMENT OF PUBLIC HEALTH

6th December, 2021

Office of the Assistant DHO Environmental Health 17 JAN 2022 SHEEMA DISTRICT

Incharge Accord this MPH student necessary support.

TO WHOM IT MAY CONCERN

RE: ODONGO DICKENS **REG. NO:** 19/HDO3/U/0109

This is to introduce to you the above-named person, a second-year student of Lira University pursuing Master of Public Health (MPH) Degree.

In partial fulfillment of the requirements for the award of Masters' Degree, he/she is carrying out research titled "Cost -Effectiveness of adding Tuberculosis Household Contact Investigation on Passive case finding strategy in South Western Uganda

The purpose of this letter is therefore to request you to allow him/her to access information/data from your organization. The data collected will be for academic purposes only.

We shall highly appreciate any assistance accorded to him/her during the course of the fieldwork.

Yours sincerely,

LIRA UNIVERSITY
P.O BOX 1035, LIRA
06 .12. 2021
HEAD OF DEPT.
PUBLIC HEALTH

MS AKELLO ANNE RUTH
HEAD OF DEPARTMENT, PUBLIC HEALTH

Relieved and accorded help
0774916484
19th/01/2022

Kigarama HC III — Received and allowed to do the needful. [illegible] 19/01/22

KIGARAMA HEALTH CENTRE III
P.O BOX [illegible], KASHONGI
SHEEMA DISTRICT

LIRA UNIVERSITY
THE BEACON

LIRA UNIVERSITY

P.O. Box 1035
Lira, Uganda
Tel: +256 414-694716
Web: www.lirauni.ac.ug
Email: health.faculty@lirauni.ac.ug.

FACULTY OF HEALTH SCIENCES
DEPARTMENT OF PUBLIC HEALTH

Office of the Assistant DHO Environmental Health
17 JAN 2022
SHEEMA DISTRICT

In charges, accord this MPH student necessary support. [illegible]

6th December, 2021

TO WHOM IT MAY CONCERN

RE: ODONGO DICKENS **REG. NO:** 19/HDO3/U/0109

This is to introduce to you the above-named person, a second-year student of Lira University pursuing Master of Public Health (MPH) Degree.

In partial fulfillment of the requirements for the award of Masters' Degree, he/she is carrying out research titled "Cost -Effectiveness of adding Tuberculosis Household Contact Investigation on Passive case finding strategy in South Western Uganda

The purpose of this letter is therefore to request you to allow him/her to access information/data from your organization. The data collected will be for academic purposes only.

We shall highly appreciate any assistance accorded to him/her during the course of the fieldwork.

Yours sincerely,

LIRA UNIVERSITY
P.O BOX 1035. LIRA
06 .12. 2021
HEAD OF DEPT.
PUBLIC HEALTH

MS AKELLO ANNE RUTH
HEAD OF DEPARTMENT, PUBLIC HEALTH

Kitagata Hospital

LIRA UNIVERSITY

P.O. Box 1035
Lira, Uganda
Tel: +256 414-694716
Web: www.lirauni.ac.ug
Email: health.faculty@lirauni.ac.ug.

FACULTY OF HEALTH SCIENCES
DEPARTMENT OF PUBLIC HEALTH

6th December, 2021

Office of the Assistant DHO Environmental Health 17 JAN 2022 SHEEMA DISTRICT

In charge, accord this MPH student necessary support.

TO WHOM IT MAY CONCERN

RE: ODONGO DICKENS **REG. NO:** 19/HDO3/U/0109

This is to introduce to you the above-named person, a second-year student of Lira University pursuing Master of Public Health (MPH) Degree.

In partial fulfillment of the requirements for the award of Masters' Degree, he/she is carrying out research titled "Cost -Effectiveness of adding Tuberculosis Household Contact Investigation on Passive case finding strategy in South Western Uganda

The purpose of this letter is therefore to request you to allow him/her to access information/data from your organization. The data collected will be for academic purposes only.

We shall highly appreciate any assistance accorded to him/her during the course of the fieldwork.

Yours sincerely,

LIRA UNIVERSITY P.O BOX 1035, LIRA 06.12.2021 HEAD OF DEPT. PUBLIC HEALTH

MS AKELLO ANNE RUTH
HEAD OF DEPARTMENT, PUBLIC HEALTH

Received and accepted.

Medical Superintendent Kitagata Hospital Sheema District Local Gov't P.O. Box 160, Kabwohe

GULU UNIVERSITY

P.O. Box 166, Gulu (U)
Website: www.gu.ac.ug
Email: gurec@gu.ac.ug

Tel: +256 471 432 096
Fax: +256 471 432 913
Mob: +256 772 305 621
+256 776 812 147

RESEARCH ETHICS COMMITTEE

16/11/2021

To: Dickens Odongo

Lira University
0758246030

Type: Initial Review

Re: GUREC-2021-100: Cost-Effectiveness of adding Tuberculosis Household Contact Investigation on Passive Case Finding strategy in South Western Uganda, PDF, 3.0, 2021-11-12

I am pleased to inform you that at the **77th** convened meeting on **23/09/2021**, the Gulu University REC, committee meeting, etc voted to approve the above referenced application.
Approval of the research is for the period of **16/11/2021** to **16/11/2022.**

As Principal Investigator of the research, you are responsible for fulfilling the following requirements of approval:

1. All co-investigators must be kept informed of the status of the research.
2. Changes, amendments, and addenda to the protocol or the consent form must be submitted to the REC for re-review and approval **prior** to the activation of the changes.
3. Reports of unanticipated problems involving risks to participants or any new information which could change the risk benefit: ratio must be submitted to the REC.
4. Only approved consent forms are to be used in the enrollment of participants. All consent forms signed by participants and/or witnesses should be retained on file. The REC may conduct audits of all study records, and consent documentation may be part of such audits.
5. Continuing review application must be submitted to the REC **eight weeks** prior to the expiration date of **16/11/2022** in order to continue the study beyond the approved period. Failure to submit a continuing review application in a timely fashion may result in suspension or termination of the study.
6. The REC application number assigned to the research should be cited in any correspondence with the REC of record.
7. You are required to register the research protocol with the Uganda National Council for Science and Technology (UNCST) for final clearance to undertake the study in Uganda.

The following is the list of all documents approved in this application by Gulu University REC:

DISTRICT HEALTH OFFICER
07 DEC 2021
NTUNGAMO

GULU UNIVERSITY
INSTITUTIONAL REVIEW COMMITTEE
APPROVED UNTIL
FACULTY OF MEDICINE
P. O. Box 166, Gulu

Received and recc
ended to carryout
Research
Bridget Ag ADHO
To I/c Burongye
H/c III

GULU UNIVERSITY

P.O. Box 166, Gulu (U)
Website: www.gu.ac.ug
Email: gurec@gu.ac.ug

Tel: +256 471 432 096
Fax: +256 471 432 913
Mob: +256 772 305 621
+256 776 812 147

RESEARCH ETHICS COMMITTEE

Received [illegible]
NGOMA H/CENTRE
P.O. NTUNGAMO
DATE 8/12/2021

16/11/2021

To: Dickens Odongo

Lira University
0758246030

Type: Initial Review

Re: GUREC-2021-100: Cost-Effectiveness of adding Tuberculosis Household Contact Investigation on Passive Case Finding strategy in South Western Uganda, PDF, 3.0, 2021-11-12

I am pleased to inform you that at the **77th** convened meeting on **23/09/2021**, the Gulu University REC, committee meeting, etc voted to approve the above referenced application.
Approval of the research is for the period of **16/11/2021** to **16/11/2022.**

As Principal Investigator of the research, you are responsible for fulfilling the following requirements of approval:

1. All co-investigators must be kept informed of the status of the research.
2. Changes, amendments, and addenda to the protocol or the consent form must be submitted to the REC for re-review and approval **prior** to the activation of the changes.
3. Reports of unanticipated problems involving risks to participants or any new information which could change the risk benefit: ratio must be submitted to the REC.
4. Only approved consent forms are to be used in the enrollment of participants. All consent forms signed by participants and/or witnesses should be retained on file. The REC may conduct audits of all study records, and consent documentation may be part of such audits.
5. Continuing review application must be submitted to the REC **eight weeks** prior to the expiration date of **16/11/2022** in order to continue the study beyond the approved period. Failure to submit a continuing review application in a timely fashion may result in suspension or termination of the study.
6. The REC application number assigned to the research should be cited in any correspondence with the REC of record.
7. You are required to register the research protocol with the Uganda National Council for Science and Technology (UNCST) for final clearance to undertake the study in Uganda.

The following is the list of all documents approved in this application by Gulu University REC:

DISTRICT HEALTH OFFICER
07 DEC 2021
NTUNGAMO

GULU UNIVERSITY
INSTITUTIONAL REVIEW COMMITTEE
APPROVED UNTIL
FACULTY OF MEDICINE
P. O. Box 166, Gulu

Recieved and Recommended to carry out the research
[illegible] Ag. ADHO
7/12/202[illegible]
To I/C Ngoma H/C [illegible]

GULU UNIVERSITY

P.O. Box 166, Gulu (U)
Website: www.gu.ac.ug
Email: gurec@gu.ac.ug

Tel: +256 471 432 096
Fax: +256 471 432 913
Mob: +256 772 305 621
+256 776 812 147

RESEARCH ETHICS COMMITTEE

To: Dickens Odongo **16/11/2021**

Lira University
0758246030

Type: Initial Review

Re: GUREC-2021-100: Cost-Effectiveness of adding Tuberculosis Household Contact Investigation on Passive Case Finding strategy in South Western Uganda, PDF, 3.0, 2021-11-12

I am pleased to inform you that at the **77th** convened meeting on **23/09/2021**, the Gulu University REC, committee meeting, etc voted to approve the above referenced application.
Approval of the research is for the period of **16/11/2021** to **16/11/2022.**

As Principal Investigator of the research, you are responsible for fulfilling the following requirements of approval:

1. All co-investigators must be kept informed of the status of the research.
2. Changes, amendments, and addenda to the protocol or the consent form must be submitted to the REC for re-review and approval **prior** to the activation of the changes.
3. Reports of unanticipated problems involving risks to participants or any new information which could change the risk benefit: ratio must be submitted to the REC.
4. Only approved consent forms are to be used in the enrollment of participants. All consent forms signed by participants and/or witnesses should be retained on file. The REC may conduct audits of all study records, and consent documentation may be part of such audits.
5. Continuing review application must be submitted to the REC **eight weeks** prior to the expiration date of **16/11/2022** in order to continue the study beyond the approved period. Failure to submit a continuing review application in a timely fashion may result in suspension or termination of the study.
6. The REC application number assigned to the research should be cited in any correspondence with the REC of record.
7. You are required to register the research protocol with the Uganda National Council for Science and Technology (UNCST) for final clearance to undertake the study in Uganda.

The following is the list of all documents approved in this application by Gulu University REC:

DISTRICT HEALTH OFFICER
07 DEC 2021
NTUNGAMO

GULU UNIVERSITY
INSTITUTIONAL REVIEW COMMITTEE
APPROVED UNTIL
[illegible]
FACULTY OF MEDICINE
P. O. Box 166, Gulu

Recieved and Recommended to carry out the research
[signature] Ag. ADHO-MCH
7/12/2021

To I/C Kihwe HC IV
[signature]

GULU UNIVERSITY

P.O. Box 166, Gulu (U)
Website: www.gu.ac.ug
Email: gurec@gu.ac.ug

Tel: +256 471 432 096
Fax: +256 471 432 913
Mob: +256 772 305 621
+256 776 812 147

RESEARCH ETHICS COMMITTEE

To: Dickens Odongo

16/11/2021

Lira University
0758246030

Type: Initial Review

Re: GUREC-2021-100: Cost-Effectiveness of adding Tuberculosis Household Contact Investigation on Passive Case Finding strategy in South Western Uganda, PDF, 3.0, 2021-11-12

I am pleased to inform you that at the **77th** convened meeting on **23/09/2021**, the Gulu University REC, committee meeting, etc voted to approve the above referenced application.
Approval of the research is for the period of **16/11/2021** to **16/11/2022.**

As Principal Investigator of the research, you are responsible for fulfilling the following requirements of approval:

1. All co-investigators must be kept informed of the status of the research.
2. Changes, amendments, and addenda to the protocol or the consent form must be submitted to the REC for re-review and approval **prior** to the activation of the changes.
3. Reports of unanticipated problems involving risks to participants or any new information which could change the risk benefit: ratio must be submitted to the REC.
4. Only approved consent forms are to be used in the enrollment of participants. All consent forms signed by participants and/or witnesses should be retained on file. The REC may conduct audits of all study records, and consent documentation may be part of such audits.
5. Continuing review application must be submitted to the REC **eight weeks** prior to the expiration date of **16/11/2022** in order to continue the study beyond the approved period. Failure to submit a continuing review application in a timely fashion may result in suspension or termination of the study.
6. The REC application number assigned to the research should be cited in any correspondence with the REC of record.
7. You are required to register the research protocol with the Uganda National Council for Science and Technology (UNCST) for final clearance to undertake the study in Uganda.

The following is the list of all documents approved in this application by Gulu University REC:

DISTRICT HEALTH OFFICE
07 DEC 2021
NTUNGAMO

GULU UNIVERSITY
INSTITUTIONAL REVIEW COMMITTEE
APPROVED UNTIL
[illegible]
FACULTY OF MEDICINE
P. O. Box 166, Gulu

Recieved and Recommended to carryout the Research.
[illegible] Ag. ADHO-MCH
To I/c Rushooka HC III.

DHO - copy

LIRA UNIVERSITY

P.O. Box 1035
Lira, Uganda
Tel: +256 414-694716
Web: www.lirauni.ac.ug
Email: health.faculty@lirauni.ac.ug.

FACULTY OF HEALTH SCIENCES

DEPARTMENT OF PUBLIC HEALTH

6th December, 2021

Office of the Assistant DHO Environmental Health 17 JAN 2022 SHEEMA DISTRICT

In charge Record this MPH student necessary support

TO WHOM IT MAY CONCERN

RE: ODONGO DICKENS **REG. NO:** 19/HDO3/U/0109

This is to introduce to you the above-named person, a second-year student of Lira University pursuing Master of Public Health (MPH) Degree.

In partial fulfillment of the requirements for the award of Masters' Degree, he/she is carrying out research titled "Cost -Effectiveness of adding Tuberculosis Household Contact Investigation on Passive case finding strategy in South Western Uganda

The purpose of this letter is therefore to request you to allow him/her to access information/data from your organization. The data collected will be for academic purposes only.

We shall highly appreciate any assistance accorded to him/her during the course of the fieldwork.

Yours sincerely,

LIRA UNIVERSITY P.O BOX 1035, LIRA 06.12.2021 HEAD OF DEPT. PUBLIC HEALTH

MS AKELLO ANNE RUTH
HEAD OF DEPARTMENT, PUBLIC HEALTH

Bugongi HC III

Forwarded

LIRA UNIVERSITY

P.O. Box 1035
Lira, Uganda
Tel: +256 414-694716
Web: www.lirauni.ac.ug
Email: health.faculty@lirauni.ac.ug.

FACULTY OF HEALTH SCIENCES

DEPARTMENT OF PUBLIC HEALTH

6th December, 2021

Office of the Assistant DHO
17 JAN 2022
SHEEMA DISTRICT

Incharge
Accord this MPH student necessary support.

TO WHOM IT MAY CONCERN

RE: ODONGO DICKENS **REG. NO:** 19/HDO3/U/0109

This is to introduce to you the above-named person, a second-year student of Lira University pursuing Master of Public Health (MPH) Degree.

In partial fulfillment of the requirements for the award of Masters' Degree, he/she is carrying out research titled "Cost -Effectiveness of adding Tuberculosis Household Contact Investigation on Passive case finding strategy in South Western Uganda

The purpose of this letter is therefore to request you to allow him/her to access information/data from your organization. The data collected will be for academic purposes only.

We shall highly appreciate any assistance accorded to him/her during the course of the fieldwork.

Yours sincerely,

LIRA UNIVERSITY
P.O BOX 1035, LIRA
06 .12. 2021
HEAD OF DEPT.
PUBLIC HEALTH

MS AKELLO ANNE RUTH
HEAD OF DEPARTMENT, PUBLIC HEALTH

LIRA UNIVERSITY

P.O. Box 1035
Lira, Uganda
Tel: +256 414-694716
Web: www.lirauni.ac.ug
Email: health.faculty@lirauni.ac.ug.

FACULTY OF HEALTH SCIENCES
DEPARTMENT OF PUBLIC HEALTH

6th December, 2021

TO WHOM IT MAY CONCERN

RE: ODONGO DICKENS **REG. NO:** 19/HDO3/U/0109

This is to introduce to you the above-named person, a second-year student of Lira University pursuing Master of Public Health (MPH) Degree.

In partial fulfillment of the requirements for the award of Masters' Degree, he/she is carrying out research titled "Cost -Effectiveness of adding Tuberculosis Household Contact Investigation on Passive case finding strategy in South Western Uganda

The purpose of this letter is therefore to request you to allow him/her to access information/data from your organization. The data collected will be for academic purposes only.

We shall highly appreciate any assistance accorded to him/her during the course of the fieldwork.

Yours sincerely,

LIRA UNIVERSITY
P.O BOX 1035, LIRA
06 .12. 2021
HEAD OF DEPT.
PUBLIC HEALTH

DISTRICT HEALTH OFFICER
RWAMPARA DISTRICT
LOCAL GOVERNMENT

MS AKELLO ANNE RUTH
HEAD OF DEPARTMENT, PUBLIC HEALTH

The officer is allowed to access Kinoni and Bugamba HC IVs to allow his study.
Dr. Twesigomwe
29/12/2021

GULU UNIVERSITY

P.O. Box 166, Gulu (U)
Website: www.gu.ac.ug
Email: gurec@gu.ac.ug

Tel: +256 471 432 096
Fax: +256 471 432 913
Mob: +256 772 305 621
+256 776 812 147

RESEARCH ETHICS COMMITTEE

16/11/2021

To: Dickens Odongo

Lira University
0758246030

Type: Initial Review

Re: GUREC-2021-100: Cost-Effectiveness of adding Tuberculosis Household Contact Investigation on Passive Case Finding strategy in South Western Uganda, PDF, 3.0, 2021-11-12

I am pleased to inform you that at the **77th** convened meeting on **23/09/2021**, the Gulu University REC, committee meeting, etc voted to approve the above referenced application.
Approval of the research is for the period of **16/11/2021** to **16/11/2022.**

As Principal Investigator of the research, you are responsible for fulfilling the following requirements of approval:

1. All co-investigators must be kept informed of the status of the research.
2. Changes, amendments, and addenda to the protocol or the consent form must be submitted to the REC for re-review and approval **prior** to the activation of the changes.
3. Reports of unanticipated problems involving risks to participants or any new information which could change the risk benefit: ratio must be submitted to the REC.
4. Only approved consent forms are to be used in the enrollment of participants. All consent forms signed by participants and/or witnesses should be retained on file. The REC may conduct audits of all study records, and consent documentation may be part of such audits.
5. Continuing review application must be submitted to the REC **eight weeks** prior to the expiration date of **16/11/2022** in order to continue the study beyond the approved period. Failure to submit a continuing review application in a timely fashion may result in suspension or termination of the study.
6. The REC application number assigned to the research should be cited in any correspondence with the REC of record.
7. You are required to register the research protocol with the Uganda National Council for Science and Technology (UNCST) for final clearance to undertake the study in Uganda.

The following is the list of all documents approved in this application by Gulu University REC:

GULU UNIVERSITY
INSTITUTIONAL REVIEW COMMITTEE
APPROVED UNTIL
★ [illegible] ★
FACULTY OF MEDICINE
P. O. Box 166, Gulu

LIRA UNIVERSITY

LIRA UNIVERSITY
THE BEACON

P.O. Box 1035
Lira, Uganda
Tel: +256 414-694716
Web: **www.lirauni.ac.ug**
Email: **health.faculty@lirauni.ac.ug.**

FACULTY OF HEALTH SCIENCES
DEPARTMENT OF PUBLIC HEALTH

6th December, 2021

TO WHOM IT MAY CONCERN

RE: ODONGO DICKENS **REG. NO:** 19/HDO3/U/0109

This is to introduce to you the above-named person, a second-year student of Lira University pursuing Master of Public Health (MPH) Degree.

In partial fulfillment of the requirements for the award of Masters' Degree, he/she is carrying out research titled "Cost -Effectiveness of adding Tuberculosis Household Contact Investigation on Passive case finding strategy in South Western Uganda

The purpose of this letter is therefore to request you to allow him/her to access information/data from your organization. The data collected will be for academic purposes only.

We shall highly appreciate any assistance accorded to him/her during the course of the fieldwork.

Yours sincerely,

LIRA UNIVERSITY
P.O BOX 1035, LIRA
06.12.2021
HEAD OF DEPT.
PUBLIC HEALTH

MS AKELLO ANNE RUTH
HEAD OF DEPARTMENT, PUBLIC HEALTH

BUGAMBA HC IV RWAMPARA
DATE 5/1/2022

The Participant is allowed to access Bugamba HCIV for his research study. Dr [illegible]

BUGAMBA HC IV RWAMPARA
