# Supplementary material for: Cost-effectiveness analysis of adding tuberculosis household contact investigation on passive case-finding strategy in Southwestern Uganda
Source: PLoS One. 2023 Dec 21;18(12):e0288761. doi: 10.1371/journal.pone.0288761 (PMC10735033; doi:10.1371/journal.pone.0288761)
Supplement: Attachment — Submitted filename: Response to Reviewers.pdf [file pone.0288761.s009.pdf]

Lira University, Lira City West Division, Uganda.
P.O. Box 1035, Lira City
Email: ***dodongo1980@gmail.com***
Tel. ***+256 774377545, +256 785246030***
Date: 23rd June 2023

A rebuttal letter that responds to each point raised by the academic editor and reviewer(s).

1. Please ensure that your manuscript meets PLOS ONE's style requirements, including those for file naming. ***We have taken care of this throughout the manuscript****.*
2. We note that you have stated that you will provide repository information for your data at acceptance. Should your manuscript be accepted for publication, we will hold it until you provide the relevant accession numbers or DOIs necessary to access your data. If you wish to make changes to your Data Availability statement, please describe these changes in your cover letter and we will update your Data Availability statement to reflect the information you provide.
   We have made changes to the cover letter, repository information of my data can obtain after seeking a written permission from the Assistance Commissioner of the Uganda National Tuberculosis and Leprosy program, Kampala Uganda.

   Reviewers' comments:

Reviewer's Responses to Questions and Responses from the Author

Comments to the Author

1. Is the manuscript technically sound, and do the data support the conclusions?

The manuscript must describe a technically sound piece of scientific research with data that supports the conclusions. Experiments must have been conducted rigorously, with appropriate controls, replication, and sample sizes. The conclusions must be drawn appropriately based on the data presented.

Reviewer #1: Partly

Reviewer #2: Yes

---

2. Has the statistical analysis been performed appropriately and rigorously?

Reviewer #1: Yes

Reviewer #2: Yes

---

3. Have the authors made all data underlying the findings in their manuscript fully available?

The PLOS Data policy requires authors to make all data underlying the findings described in their manuscript fully available without restriction, with rare exception (please refer to the Data Availability Statement in the manuscript PDF file). The data should be provided as part of the manuscript or its supporting information or deposited to a public repository. For example, in addition to summary statistics, the data points behind means, medians and variance measures should be available. If there are restrictions on publicly sharing data—e.g., participant privacy or use of data from a third party—those must be specified.

Reviewer #1: Yes

Reviewer #2: Yes

---

4. Is the manuscript presented in an intelligible fashion and written in standard English?

PLOS ONE does not copyedit accepted manuscripts, so the language in submitted articles must be clear, correct, and unambiguous. Any typographical or grammatical errors should be corrected at revision, so please note any specific errors here.

Reviewer #1: Yes

Reviewer #2: Yes

5. Review Comments to the Author

Please use the space provided to explain your answers to the questions above. You may also include additional comments for the author, including concerns about dual publication, research ethics, or publication ethics. (Please upload your review as an attachment if it exceeds 20,000 characters)

Reviewer #1: Manuscript does not meet style/formatting guidelines of the journal. Pages are landscape and do not contain line numbers. ***We have corrected the style/formatting guidelines of the manuscript; pages are in portrait and has line numbers.***

“It entails screening for Tuberculosis infection, with or without latent TB infection” confusing wording. I believe the guidelines say that contacts do not need confirmation of a TB infection to receive TPT. ***It entails screening for Tuberculosis disease, with or without latent TB infection.” corrected.***

The analysis of is well described and clearly presented. However, the finding needs to be more nuanced. The authors note that reductions in TB transmission (and thus future TB burden) are not accounted for in their costing analysis; these are tangible benefits which are being disregarded. This results in underestimation of effectiveness, which is acknowledged as a limitation. At the same time, the negative consequences for low TB case detection in the PCF arm are not factored into the analysis. People with TB who are not treated, or only treated after a long delay, contribute to sustained burden, morbidity, and mortality. Ignoring these consequences in the PCF costs again underestimates the cost effectiveness of HCI. This is not acknowledged anywhere in the manuscript. Thus, it feels very difficult for the review to accept "adding [HCI] to the [PCF] in the Southwestern Uganda Tuberculosis control program was not cost-effective". ***We have included the comments in the discussion section (page 22) and acknowledge them in the limitation***. ***We did not factor some of the long-term effects of HCI (Death Averted, Life saved, reduction of burden of TB, transmission) because of short analytical time horizon of this study***. in addition, the discussion makes no comment on the lack of TPT provision, which should have major implications for TB burden (cases averted) and HIV epidemic control in the medium and long term. Costs for TPT provision can be subsidized through HCI. They also do not comment on the entry point for contact investigation – TB symptoms – which could result in about 40% of people with infectious pulmonary TB being missed (according to Uganda prevalence survey findings). ***We appreciated these comments and has been included in our manuscript, Discussion section page 22.*** Given that HCI is one of the most basic packages of services in TB care, I recommend the authors word their findings more carefully. Something like, this specific implementation of PCF+HCI proved to be not cost-effective, rather than HCI as a strategy. ***We have taken this recommendation seriously and included in the Conclusion section of the manuscript and abstract***. There is some context for the analysis's limitations in the discussion, but strongly worded sentences are included elsewhere which can overstate the findings.

Reviewer #2: While the abstract is clearly presented by the authors, they miss out on key pieces of information required for a cost effectiveness study. For example, in the abstract there is no clear mention if any discounting rates were applied in this study, to allow readers to understand the implications of such decisions in the analysis. ***We did not apply a discount rate because of the short analytic time horizon***. ***This was mentioned in the abstract page 2 and methodology section page 7 of the manuscript***. it was also critical for the authors to clearly state how the sensitivity analysis was used to address uncertainty. **It was stated in the result section of page 12 and Discussion section of page 21 of the manuscript.**

The methods and results sections are comprehensive, and the authors should be applauded for sharing the key ingredients of data that went into this analysis. However, it is critical for the authors to mention if this was a synthesis-based evaluation of already existing data and to which extent this data was used. This can be mentioned in the abstract. ***It was a synthesis-based evaluation of already existing data and data was extracted from the national system, as well as from the available primary tools at the facility. We mentioned it in the abstract and methodology section.***
